# Numerical Prediction of the Effect of Laser Shock Peening on Residual Stress and Fatigue Life of Ti-6Al-4V Titanium Alloy

**DOI:** 10.3390/ma15165503

**Published:** 2022-08-10

**Authors:** Peixuan Ouyang, Xuekun Luo, Zhichao Dong, Shuting Zhang

**Affiliations:** 1 School of Mechanical and Material Engineering, North China University of Technology, Beijing 100144, China; 2 AECC Beijing Institute of Aeronautical Materials, Beijing 100095, China

**Keywords:** laser shock peening, fatigue life, four-point bend, compressive residual stress, numerical prediction

## Abstract

Laser shock peening (LSP) is a promising surface strengthening technology to improve the fatigue life of alloy components. In this work, the residual stress field of Ti-6Al-4V titanium alloy induced by LSP was simulated based on finite element method (FEM), and then the fatigue lives of the non-LSP and LSP-treated specimens subjected to four-point bending were predicted using the software Fe-safe. The simulation results were compared and validated with the corresponding experimental results. LSP treatment produces a maximum compressive residual stress (CRS) of up to 800 MPa on the surface of the specimen and a CRS layer with a thickness of 0.623 mm under the upper surface of the specimen. The existence of the CRS layer reduces the maximum principal stress from 608 MPa to 540 MPa and changes its location from the upper surface into the internal position at a depth of about 0.6 mm during the four-point bending process. This results in experimental and predicted fatigue lives 4.2 and 17.24 times longer for the specimens with LSP pretreatment compared to those without LSP.

## 1. Introduction

Fatigue life is a key performance indicator for titanium alloy components serving under cycle or vibration loadings in applications in the aviation, aerospace and other industries [1,2]. Many mechanical surface processing methods, such as laser shock peening (LSP) [3,4], shot peening (SP) [5,6], cold extruding [7,8] and deep rolling [5,9], have been developed to prolong fatigue life. 

LSP is a prospective surface strengthening technology using laser-induced shockwaves to bring in a near-surface layer of beneficial compressive residual stress (CRS) with a thickness of about 0.5~2 mm, improving the anti-fatigue performance [10,11,12]. The CRS induced by LSP is generally larger and extends to a greater depth, leading to a superior fatigue life compared with SP [13]. Both the magnitude and the distribution of the LSP-induced residual stress play a vital part in enhancing fatigue performance of the components since they have decisive influences on crack initiation and propagation [14,15]. Significant experimental works have been carried out to study the effect of LSP and its parameters on the generated CRS and fatigue life for many alloys [16,17,18,19]. Meanwhile, numerical simulations on the LSP-induced CRS distribution have been conducted to establish correlations between the stress distribution and different processing parameters, since finite element method (FEM) could provide a more clear and comprehensive study of the residual stress field distribution than measurement methods such as XRD or hole drilling. Xiang et al. [20] studied the effect of three critical LSP parameters—scanning patterns, overlapping rate and laser spot shape—on the residual stress and displacement deformation of 7075 aluminum alloy by FEM. Adu-Gyamfi et al. [21] simulated the effect of laser scanning patterns on residual stress distribution and carried out fatigue test of Al 2024-T351 specimens. Correa et al. [22] studied the effect of advancing direction on the fatigue life of 316 L stainless steel by simulating the induced residual stress field and performing fatigue experiment. However, most previous numerical simulation studies have only focused on the CRS magnitude and distribution induced by LSP, and the effect of the LSP-induced CRS layer on the fatigue stress distribution and fatigue life is less studied by the numerical simulation method, which has several advantages compared to the experimental method. On one hand, since it is much easier to observe the evolution of stress distribution using numerical simulation compared with on the basis of stress measurement during fatigue loading, the inner relationship between LSP-induced stress distribution and fatigue performance can be better elucidated by means of simulation. On the other hand, numerical simulations save plenty of time and financial resources compared to fatigue tests. Therefore, it would be a promising study to combine the simulation of LSP-induced residual stress and the prediction of fatigue life to efficiently promote the understanding of the mechanism of influence of LSP treatment on fatigue performance. 

In this paper, the effect of LSP treatment on the induced residual stress distribution and fatigue life of Ti-6Al-4V titanium alloy coupon under four-point bend loading condition were investigated with numerical simulation method. The simulation results were compared and validated with experimental data. This study achieves a connection between the LSP-induced residual stress simulation and the fatigue performance prediction, which would provide a convenient, rapid and effective mean to better understand the effect of LSP processes on fatigue life for specific applications and provide optimal use of LSP technology.

## 2. Experimental Section

### 2.1. LSP Process and Stress Characterization

**Figure 1:**
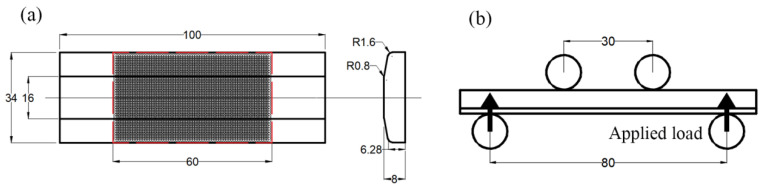
(**a**) Dimensions of the specimen with the illustration of laser peened region; (**b**) schematic diagram of four-point bend fatigue test set-up.

The specimens studied in this study were Ti-6Al-4V titanium alloy coupons with an overall length of 100 mm, a width of 34 mm, and a maximum thickness of 8 mm. Before LSP and the four-point bend fatigue test, the surface of the specimens were ground to the roughness Ra of about 0.825 μm, producing a surface CRS of 53 MPa. The two surfaces of the specimen were peened alternately in turn, with an area of 60 mm × 34 mm indicated by the red dotted rectangle in Figure 1a, which is the same as in our previous work [16]. A Q-switched Nd:YAG pulse laser with a near-Gaussian temporal profile was employed with a wavelength of 1064 nm, a pulse duration of 10 ns at FWHM (Full Width at Half Maximum), pulse energy of 2.4 J and beam diameter of 1.8 mm for laser peening experiment. The laser scanning pattern was a zigzag type with an overlapping rate of 50% and the advancing direction was parallel to the width of the specimen. The flow water was adopted as the confinement layer and the black tape was adopted as the absorbent layer. One end of the samples was clamped by a machine hand to move in relation to the fixed laser beam. The residual stresses of the specimens after LSP were measured by XRD diffraction with Cu Kα ray. The specimens were removed layer by layer via electrolytic polishing to obtain the depth profile of the residual stress.

### 2.2. Fatigue Test

The non-LSP-treated and LSP-treated specimens were subjected to a four-point bend fatigue test on an MTS 50 kN universal test machine in open air at room temperature. A schematic diagram of the experimental set-up is presented in Figure 1b. There were two parallel load rods keeping a distance of 80 mm, and two supporting rods keeping a distance of 30 mm on the opposite surface. Constant amplitude cyclic loads with a maximum stress of 550 MPa were applied in a sinusoidal waveform at a frequency of 5 Hz and a stress ratio of R = 0.1.

## 3. Numerical Modeling

**Figure 2:**
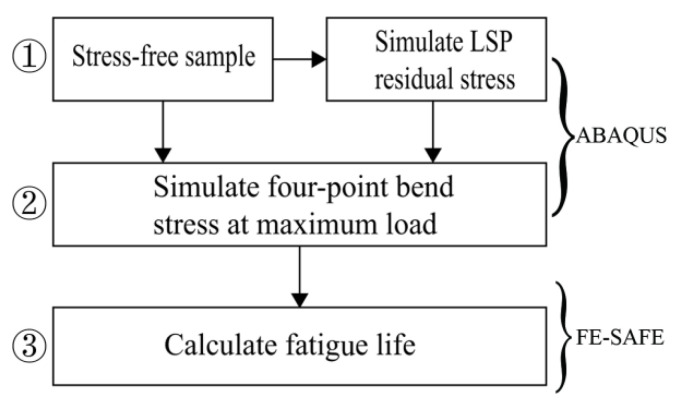
Framework of numerical modeling.

The numerical modeling framework in this study included three steps, as shown in Figure 2. The first step was to simulate the LSP-induced residual stress distribution of the specimen using the commercial FEA software ABAQUS/Explicit. ABAQUS/Explicit is suitable for solving nonlinear dynamic problems and quasi-static problems, especially for simulating short, instantaneous dynamic events such as shocks and explosions. The second step was to calculate the stress states of both the non-LSP-treated and LSP-treated specimens subjected to four-point bending under the maximum loading stress by ABAQUS/Standard. ABAQUS/Standard is capable of solving a wide range of linear and nonlinear problems, including static analysis and dynamic analysis. The last step was to calculate the fatigue lives of the specimens using ANSYS FE-SAFE, which is recognized as the most accurate fatigue analysis software in the world with advanced algorithms, comprehensive and detailed functions.

### 3.1. LSP Process and Stress Characterization

Laser peening is generally modeled as a mechanical process [23]. Due to the evolution of laser energy with time and space, the pressure of laser-induced shock wave is a function of time and space as follows: P(x, y, t) = P_max_·P(t)·P(x, y). The peak pressure of the shock wave P_max_ was estimated by the formula proposed by F. Fabbro [24], where α is the ratio of internal energy to heat energy and generally taken as 0.1~0.2 [25], Z is equivalent acoustic impedance and defined as Z = 2/(1/Z_H2O_ + 1/Z_Ti_), Z_H2O_ and Z_Ti_ are acoustic impedances of water and titanium alloy, respectively (Z_H2O_ = 1.65 × 10^5^ g·cm^−2^·s^−1^, Z_Ti_ = 2.93 × 10^6^ g·cm^−2^·s^−1^). The laser density I_0_ was 9.436 GW/cm^2^, as calculated from the given laser parameters and the peak pressure P_max_ was taken as 3.5 GPa. The pressure–time distribution shape for the laser-induced shock wave is similar to the laser temporal profile, even though the FWHM of the pressure waveform is about 2~3 times the laser pulse duration [26,27]. For a single Gaussian laser temporal pulse, the corresponding Gaussian pressure-time profile was simplified into a triangle ramp since the Gaussian pressure profile with a very short pulse duration (order of 100 ns) is very close to a triangular ramp [28,29]. Specifically, the pressure rose linearly to reach the peak pressure in a duration of 10 ns and then linearly decreased in the following 10 ns. The spatial profile of the pressure pulse obeyed a near-spherical distribution [30]. These input data for the pressure pulse and the scanning patterns were programed using a Fortran subroutine, VDLOAD.

During the LSP process, the material undergoes deformation with extremely high strain rate exceeding 10^6^ s^−1^, so the glasto-plastic behavior of Ti-6Al-4V titanium alloy was modeled using the Johnson–Cook constitutive equation [30,31], which generally describes the strength limit and failure process of metallic materials under conditions of large strains, high strain rates and high temperatures. Since the temperature rise caused by stress wave propagation during LSP is very small (~60 K), the thermal effect can be ignored. The parameters of the constitutive equation and mechanical properties of Ti-6Al-4V titanium alloy are provided in Table 1 [31,32]. A, B, C and *n* are yield stress, work hardening modulus, strain rate sensitivity and work hardening coefficient, respectively.

**Figure 3:**
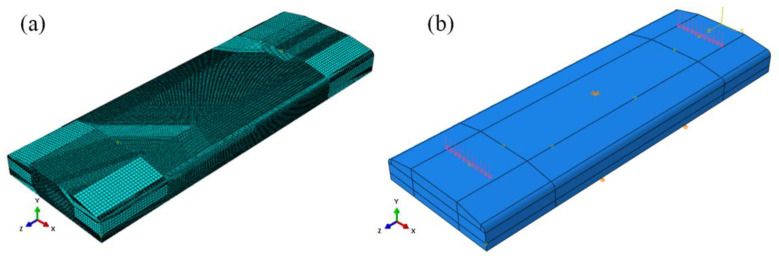
(**a**) Schematic of the 3D FEM model of specimen for LSP; (**b**) load and boundary constraints for the four-point bending specimen.

A three-dimensional geometry model of the specimen was established to study the LSP-induced residual stress, as shown in Figure 3a. Usually, elastic-plastic finite elements are used in the plastic zone to capture the residual stress field, while the only elastic infinite elements are added as non-reflective boundary conditions to model the remaining material without plastic deformation. However, since both the upper and lower regions of the specimen should suffer from plastic deformation during alternate double-sided laser peening, the infinite elements were not adopted in this work. A total of 1,835,750 elements with the element type of C3D8R (3D eight-node linear element with reduced integration and hourglass control) were used in the whole mesh. The smallest element size in the LSP treated region was 200 μm × 200 μm × 50 μm. To remain consistent with the experimental conditions, the displacements and rotations of the nodes at one end of the specimen were fully constrained.

The model was solved by the “modified-explicit” procedure [23], also known as the extended explicit method. The explicit solver ran for a short duration of 50 μs for each single pulse until kinetic energy tended to be zero and the plasticity reached saturation. For the final shot, an extended explicit simulation time of 3 ms replaced the time-consuming force equilibrium analysis in the implicit solver. In the work, artificial damping was not introduced to accelerate the dissipation of kinetic energy and shorten the calculational time, since it should be done meticulously to avoid reducing the prediction accuracy of the model. To capture the propagation of the shock wave, the incremental step time should not exceed the propagation time of the elastic wave in the finest mesh element, namely ∆t ≤ ∆t_stable_ = L_e_/C_d_, where Le is the size of the finest mesh element and C_d_ is the propagation speed of elastic wave in the material, which is determined by the formula [31], namely, C_d_ is equal to 6158.6 m/s for the Ti-6Al-4V alloy. Thus, ∆t_stable_ = 8.1 ns, and the incremental step time was taken as 4 ns. The simulation was run using parallel computation which divided the problem into several parts and calculated each part by an independent processor at the same time.

### 3.2. Four-Point Bend Stress Simulation

Four-point bending coupon models were constructed for the stress-free non-LSP specimen and the LSP specimen with the initial stress state having been calculated in Section 3.1. The grid division and element type of the models were kept the same as those in the LSP simulation. The load was applied with a uniform pressure of 550 MPa at the nodes where the coupon is in contact with two load rods, as shown in Figure 3b. Zero vertical displacement was applied on the nodes where the coupon is contact with two supporting rods. Zero displacement constraints in the X and Z axes were exerted for the central node on the upper surface.

### 3.3. Fatigue Life Simulation

The stress simulation results of four-point bending were imported into the Fe-safe fatigue analysis software to calculate fatigue lives. The Seeger material approximation algorithm in the Fe-safe material database provides a method with sufficient accuracy for estimating the fatigue performance of most materials by inputting simple tensile data such as tensile strength and elastic modulus [33]. In this way, the S-N curve of TC4 titanium alloy was obtained. In addition, the Goodman model was adopted as the fatigue life algorithm, which is relatively conservative, and is widely applied in engineering [34]. A sinusoidal waveform load spectrum was adopted with a stress ratio of R = 0.1. The load size of a cycle was controlled by setting a dataset value of (1, 0.1). The surface roughness and initial residual stress of the specimen caused by grinding was taken into account.

## 4. Results and Discussion

### 4.1. Residual Stress Induced by LSP

**Figure 4:**
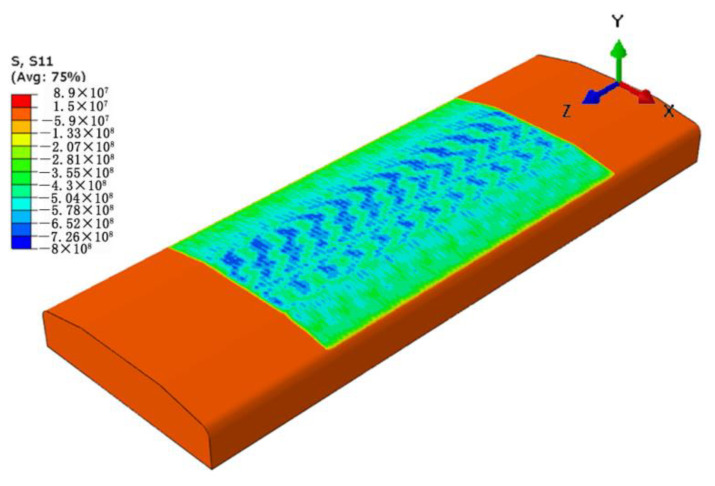
Simulated 3D residual stress filed of the LSP-treated specimen.

**Figure 5:**
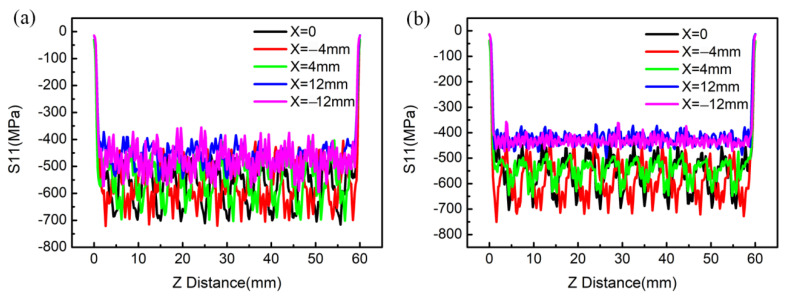
Residual stress distributions along the paths (**a**) on the upper surface and (**b**) on the lower surface.

In XRD residual stress measurements, the surface stress of a material is usually in a two-dimensional stress state and the stress in the normal direction is zero due to the small penetration depth of the X-ray. This indicates that only the stresses in the X and Z directions of the specimen (namely, S11 and S33) can be measured. Therefore, the stress S11 of the specimen induced by LSP was presented for comparison and verification with the measured stress by XRD. Figure 4 presents the simulated residual stress field of the specimen treated by LSP. The maximum CRS is up to 800 MPa. For the purpose of studying the magnitude and distribution uniformity of the surface residual stress, the surface stress was extracted along the paths being parallel to the Z direction (X = −12, −4, 0, 4, 12 mm) for both the upper and lower surfaces. Since the stresses of the five paths could also reflect the stress distribution characteristic along the paths being parallel to the X direction and their average value could reasonably reflects the stress state of the whole surface, the stress along the paths being parallel to the X direction is not extracted and presented. The stress curves of the five paths are presented in Figure 5. This shows that the CRS at the paths of X = 0, ±4 mm are higher than those at the paths of X = ±12 mm. This indicates that the CRS close to the edge of the specimen is lower than that far away from the edge of the specimen, which might be due to an edge effect. However, the fluctuations of the residual stress at the paths of X = ±12 mm are smaller than those at the paths of X = 0, ±4 mm. By averaging the residual stresses of the paths on the same surface, the average residual stresses of the upper and lower surfaces of the specimen are −546 ± 78 MPa and −561 ± 63 MPa, respectively.

**Figure 6:**
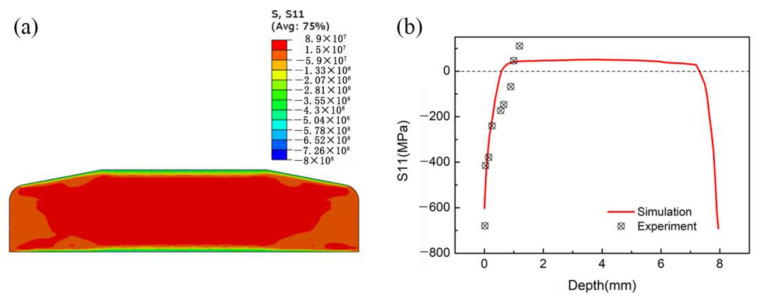
(**a**) Cross-sectional residual stress distribution of the LSP-treated specimen; (**b**) comparison of simulated and measured residual stresses along the depth of the specimen.

Figure 6a presents the cross-sectional residual stress distribution of the LSP-treated specimen. There is compressive residual stress at and close to the two surfaces, while tensile stress is present in the central region inside the specimen. Figure 6b shows the simulated and measured residual stresses along the depth of the specimen. The X-ray diffraction measurement has a high resolution in the depth direction, since X-ray penetrates only approximately 10 μm in titanium [35], which is smaller than the height of the finite element mesh (50 μm). However, the measurement resolution in the horizontal direction is not so high due to the larger irradiated beam area with the diameter of 1 mm2 compared with the size of the finite element mesh (200 μm × 200 μm) [36]. It can be seen that the simulation results fit the experimental results quite well, indicating that the numerical modeling of residual stress induced by LSP is reliable. In addition, the residual compressive stress on the upper surface is slightly lower than that on the lower surface. The depths of residual compressive stress on the upper and lower sides are 0.623 mm and 0.718 mm, respectively.

### 4.2. Four-Point Bending Stress

**Figure 7:**
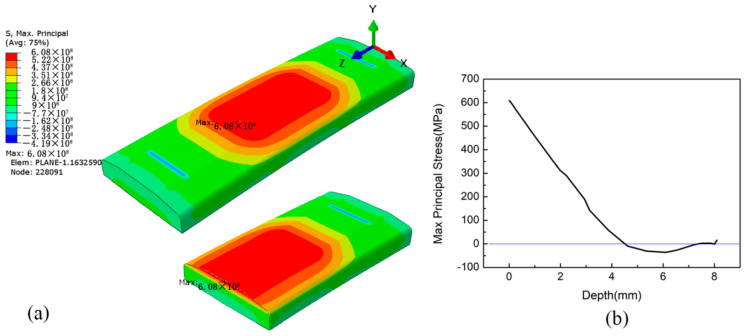
Maximum principal stress of the non-LSP specimen subjected to four-point bending: (**a**) stress field distribution; (**b**) stress curve along the depth passing through node No. 228091.

To study the effect of LSP treatment on fatigue life, the stresses induced by four-point bending of both the non-LSP and LSP-treated specimens were studied. Since fatigue failure depends more on the maximum principal stress instead of the stress components; thus, the maximum principal stress induced during four-point bending was studied for the further simulation of fatigue life. Figure 7a shows the maximum principal stress filed distribution of the non-LSP specimen subjected to four-point bending. A maximum stress of 608 MPa exists at node No. 228091, which is located at Z = 63.27 mm on the upper surface of the specimen. Actually, the stress in the specimen is nearly symmetrical along the path of Z = 50, and the stress at Z = 36.73 mm in the upper surface is close to that located at Z = 63.27 mm. The maximum principal stress curve along the specimen depth passing through node No. 228091 is presented in Figure 7b. This also indicates that the maximum principal stress is located at the upper surface of the specimen, and the stress decreases gradually along the depth direction. The stress begins to turn negative at the depth of near 4.87 mm and is lowest at the depth of 6 mm. The evolution of the stress curve can be explained by the fact that the upper surface of the specimen bears tensile stress and the lower surface bears compressive stress under four-point bend loading.

**Figure 8:**
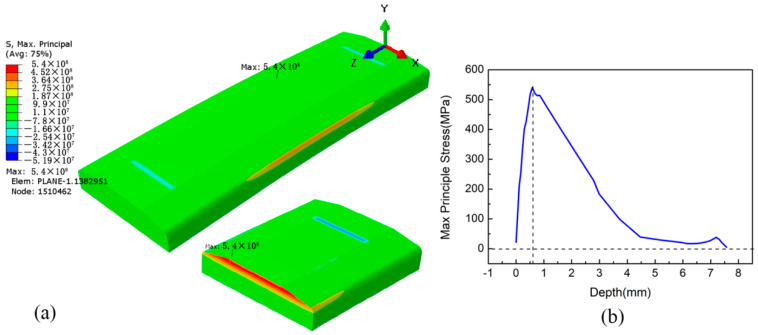
Maximum principal stress of the LSP-treated specimen subjected to four-point bending: (**a**) stress field distribution; (**b**) stress curve along the depth passing through node No. 1510462.

Figure 8a shows the maximum principal stress field distribution of the LSP-treated specimen subjected to four-point bending. It can be seen that the maximum stress of 540 MPa exists at node 1510462, which is located at Z = 39.4 mm inside the specimen instead of on the upper surface. Likewise, the stress at Z = 60.6 mm on the upper surface is close to that located at Z = 39.4 mm due to the near symmetry of stress along the path of Z = 50 mm. Figure 8b presents the maximum principal stress curve along the specimen depth passing through node No. 1510462. It can be seen that the principal stress is only about 23 MPa at the surface, and then gradually increases to reach its maximum at a depth of about 0.6 mm. After that, the stress gradually decreases to become stable at about 0 MPa. By comparing Figure 7 and Figure 8, it can be concluded that the stress distributions caused by four-point bend loading for the specimens with and without LSP treatment are quite different. The pretreatment of LSP changes the location of maximum principal stress from the upper surface into the internal position at a depth of about 0.6 mm, which is due to the CRS layer with the depth of 0.623 mm caused by LSP under the surface of the specimen. In addition, the maximum principal stress is reduced from 608 MPa to 540 MPa with the pretreatment of LSP.

### 4.3. Fatigue Life

**Figure 9:**
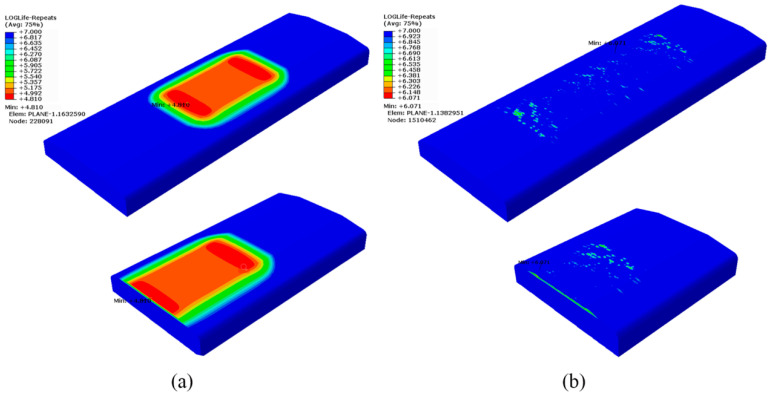
Logarithmic-form life nephograms of four-point bending fatigue: (**a**) specimen without LSP treatment; (**b**) specimen with LSP treatment.

**Figure 10:**
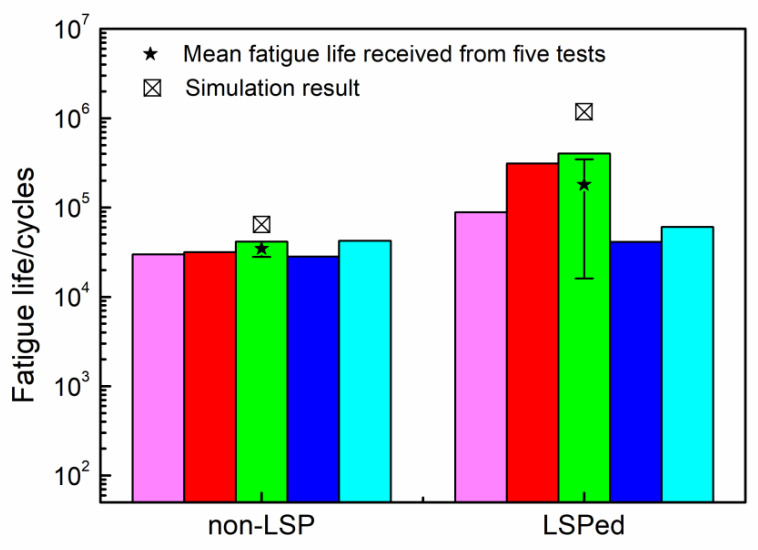
Simulated and measured fatigue lives of non-LSP and LSP-treated specimens induced by four-point bending.

Figure 9 presents logarithmic-form fatigue life nephograms of four-point bending for the specimens without and with LSP pretreatment. As shown in Figure 9a, the minimum fatigue life predicted for the non-LSP specimen is 10^4.810^ = 64,565 cycles. The corresponding position is located at node No. 228091, indicating that fatigue failure will occur preferentially at the site of Z = 63.27 mm or Z = 36.73 mm. In fact, the fatigue failure in the experiment always appears at the position of about Z = 60 mm or 40 mm. Additionally, the fatigue life obtained from the experiment using the non-LSP specimen was 34,800 cycles, as shown in Figure 10. The fatigue life predicted is only 85.5% higher than that obtained from experiment, indicating that the prediction model of fatigue life is reliable. For the LSP-treated specimen, the minimum fatigue life predicted was 10^6.071^ = 1,177,606 cycles. As shown in Figure 9b, the corresponding position is located at node No. 1510462, indicating that fatigue failure will occur preferentially at the site of Z = 39.4 mm or 60.6 mm. The fatigue life predicted is 5.5 times higher than that obtained from the experiment, which was 181,000 cycles, as shown in Figure 10. The difference between experimental and predicted fatigue lives for the specimen with LSP treatment is greater than that for the non-LSP specimen. There are two reasons for this phenomenon. On one hand, it can be seen from Figure 10 that the LSP-treated specimen has a larger fluctuation in measured fatigue life compared with the non-LSP specimen. It seems that the fatigue life of the LSP-treated specimen is more sensitive to the machining quality than that of the non-LSP specimen. If local stress concentration is induced during processing, the beneficial effect of the laser shock peening on fatigue life may not work so that the fatigue life keeps close to or slightly higher than that of the non-LSP specimen. Thus, the measured mean fatigue lives of the LSP-treated specimen might be lower than its real fatigue life. In addition, as mentioned in Section 4.1, the numerical analysis of residual stress induced by LSP has a lower resolution in the depth direction and a higher resolution in the horizontal direction compared with the measurement; thus, there are some discrepancies between the numerical and experimental results of residual stress induced by laser shock peening, as shown in Figure 6b, which could also result in discrepancies between prediction and measurement of fatigue life. For the above two reasons, the life difference for the LSP-treated specimen between prediction and experiment can be considered reasonable. For both the experimental and simulated results, the specimen with LSP treatment has a much longer fatigue life than that without LSP, namely, 4.2 and 17.24 times, respectively. This is the result of the reduced maximum principal stress and its location changing from the upper surface into the internal position induced by the CRS layer produced by LSP treatment.

Based on the above analysis, the simulation results in the residual stress distributions and the fatigue lives agree well with the experimental results, especially for the specimens without LSP treatment. Since the fatigue life of the LSP-treated specimen is more sensitive to the machining surface quality than that of the non-LSP specimen, the measured average fatigue life of the LSP-treated specimen is lower than its actual fatigue life, which is one of the main reasons for the larger difference in fatigue life between experiment and prediction. This indicates that the combination model for LSP-induced residual stress simulation and fatigue life prediction is reliable. In addition, the simulation study can not only point out the effect of the LSP treatment on the stress distribution under fatigue loading as well as the fatigue life, but can also save plenty of time and money compared with experimental study. Thus, the model in this study is expected to be applied in engineering fields to efficiently find optimal LPS process parameters allowing the components to meet service life requirements. However, there is still some work to be done in the future. On one hand, the influence of surface quality on the fatigue life measurement of LSP-treated specimens should be further avoided, in order that the beneficial effect of LSP on fatigue life can be brought into full play and more accurate lives will be measured to further validate the model. On the other hand, comparative study of fatigue lives obtained from simulations and experiments for different load amplitudes would be carried out to make the work more complete. 

## 5. Conclusions

(1) The simulation results of the residual stress field induced by LSP fit well with the experimental results. The former show that LSP treatment induces the maximum CRS on the surface of the specimen is up to 800 MPa and the thickness of the CRS layers for both the upper and lower sides are 0.623 mm and 0.718 mm, respectively.
(2) During the four-point bending process, the pretreatment of LSP reduces the maximum principal stress from 608 MPa to 540 MPa and changes the location of the maximum stress from the upper surface into the internal position at a depth of about 0.6 mm, which is due to the existence of CRS layer caused by LSP with the depth of 0.623 mm under the surface of the specimen.
(3) The fatigue lives of the specimens with LSP treatment obtained from experiment and predictions are, respectively, 4.2 and 17.24 times longer than those of the non-LSP specimens. One of the main reasons for the larger fatigue-life difference between prediction and experiment for the LSP-treated specimen compared with that for the non-LSP specimen is that the measured mean life is lower than the actual life due to the higher sensibility of fatigue life on machining quality of the LSP-treated specimen. The longer fatigue life of the LSP-treated specimen results from the reduced maximum principal stress and its location changing from the upper surface into the internal position induced by the CRS layer produced by LSP treatment.
(4) The combination model of the LSP-induced residual stress simulation and fatigue life prediction is reliable compared with the experimental results and is expected to be applied in engineering fields to efficiently find optimal LPS process parameters that make the components meet service life requirements. However, there is still some work to be done in the future. On one hand, the influence of surface quality on the fatigue life measurement of LSP-treated specimens should be further avoided so that more accurate lives will be measured to further validate the model. On the other hand, comparative study of fatigue lives obtained from simulations and experiments for different load amplitudes would be carried out to make the work more complete.

## Figures and Tables

**Table 1:** Johnson–Cook parameters and mechanical properties of Ti-6Al-4V titanium alloy.

A (MPa)	B (MPa)	*n*	C	m	${\dot{\varepsilon }}_{0}$	ρ (kg/m^3^)	E (GPa)	ν	σ_b_ (MPa)
862	331	0.34	0.012	0.8	1	4500	110	0.342	910
